# Supplementary material for: Enhanced Photocatalytic Antibacterial Properties of TiO2 Nanospheres with Rutile/Anatase Heterophase Junctions and the Archival Paper Protection Application
Source: Nanomaterials (Basel). 2021 Sep 30;11(10):2585. doi: 10.3390/nano11102585 (PMC8539383; doi:10.3390/nano11102585)
Supplement: Supplementary file 1 [file nanomaterials-11-02585-s001.zip › nanomaterials-1381693-supplementary.pdf]

Supplementary Materials

Enhanced Photocatalytic Antibacterial Properties of TiO_2 Nanospheres with Rutile/Anatase Heterophase Junctions and the Archival Paper Protection Application

Yingying Qin [1], Xinyu Wang [2], Pengyuan Qiu [2] and Jian Tian [2,*]

1 Archives Department, China University of Petroleum (East China), Qingdao 266580, China; qinyingying@upc.edu.cn
2 School of Materials Science and Engineering, Shandong University of Science and Technology, Qingdao 266590, China; skd994440@sdust.edu.cn (X.W.); skd994462@sdust.edu.cn (P.Q.)
* Correspondence: jiantian@sdust.edu.cn

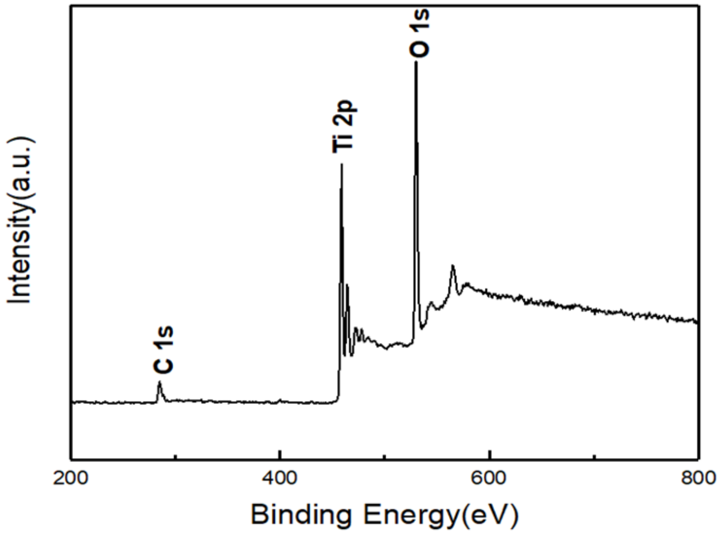

Figure S1. XPS survey spectrum of TiO_2-750.

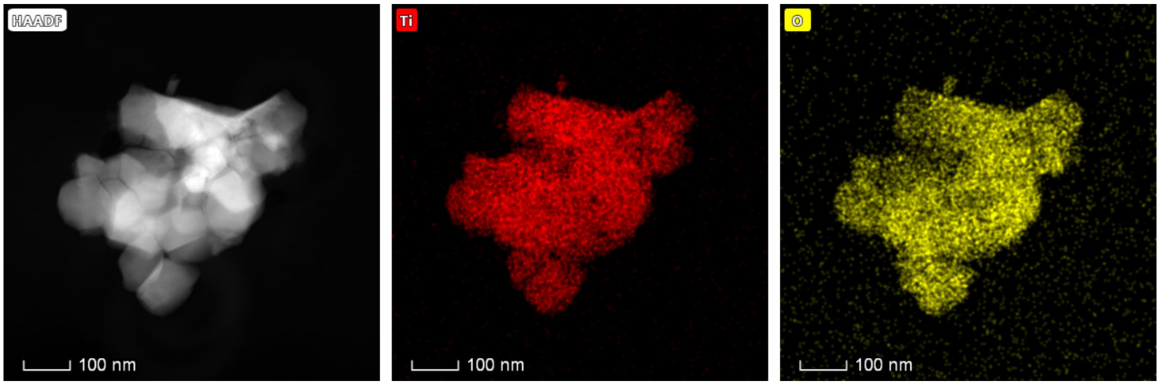

Figure S2. EDX mapping images of Ti and O in TiO_2-750.

Table S1. BET surface areas and pore sizes of TiO_2-A, TiO_2-250, TiO_2-350, TiO_2-550 and TiO_2-750.

Samples	BET Surface area ($m^2 g^{-1}$)	Pore size (nm)
TiO_2-A	16	3.6

TiO_2-250	306	3.8
TiO_2-350	302	3.8
TiO_2-550	298	4.0
TiO_2-750	297	4.2

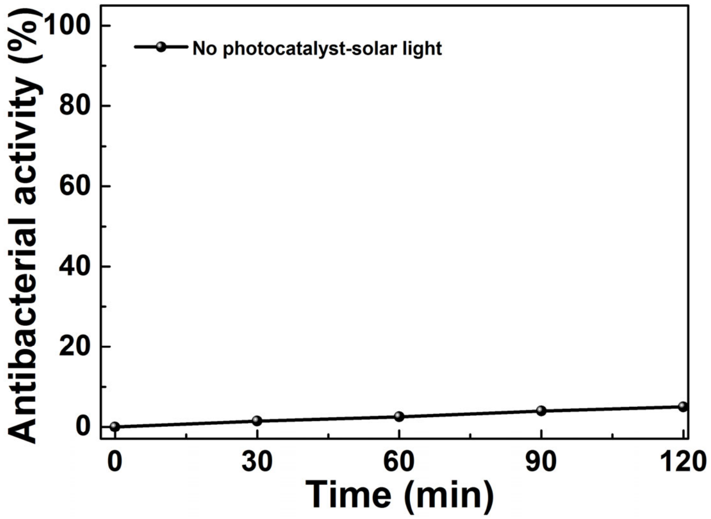

Figure S3. Photocatalytic disinfection efficiency against *E. coli* for no photocatalyst under simulated solar light irradiation.

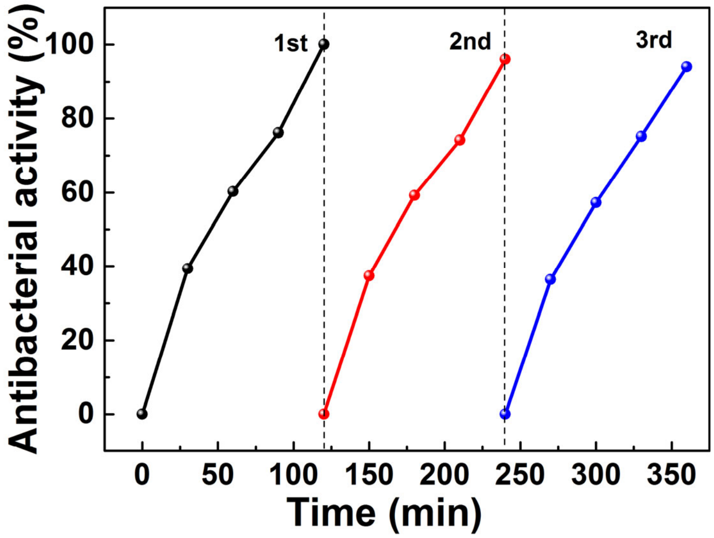

Figure S4. Photocatalytic disinfection efficiency against *E. coli* for TiO_2-750 under simulated solar light irradiation.
